# Targeting the oncogene B lymphoma deregulator IgH 3′ regulatory region does not impede the *in vivo* inflammatory response in mice

**DOI:** 10.18632/oncoscience.81

**Published:** 2014-09-19

**Authors:** Faten Saad, Alexis Saintamand, Pauline Rouaud, Yves Denizot

**Affiliations:** ^1^ CNRS UMR 7276, CRIBL, Université de Limoges, Limoges, France

**Keywords:** IgH 3′ regulatory region, knock-out mice, pristine, inflammation, cytokines

## Abstract

The IgH 3′ regulatory region (3′RR), encompassing the four transcriptional enhancers hs3a-hs1,2-hs3b-hs4, is a potent lymphoma oncogene deregulator but its role in B cell-mediated inflammatory responses is unknown. We investigated the 3′RR involvement in the *in vivo* pristane-induced inflammatory response in BALB/c mice. The lack of the 3′RR in BALB/c mice had no wide effect on the incidence, the kinetic of development and the cellular composition of peritoneal ascites. Ascite pro-inflammatory cytokines levels (IL-6, IL-21, IL-12/23, TNF-α) were unchanged while anti-inflammatory cytokines levels (IL-10, interferon-γ) were slightly increased in 3′RR-deficient BALB/c mice as compared to *wt* BALB/c mice. In conclusion, the 3′RR is dispensable for the efficient recruitment of immune cells and the normal development of an inflammatory response in the *in vivo* pristane-induced inflammatory model. The 3′RR might be considered as a potential suitable target for anti-lymphoma pharmacological therapy without potent adverse effect on normal immune and inflammatory responses.

## INTRODUCTION

The IgH locus undergoes multiple changes along B cell differentiation, affecting transcription and accessibility to V(D)J recombination, somatic hypermutation (SHM) and class switch recombination (CSR) [1, 2]. Since all Ig gene remodelling events require transcription, IgH *cis*-regulatory regions and especially transcriptional enhancers are major locus regulators. Mouse models carrying targeted genomic deletions highlights distinct roles for such regions. The *iE*μ and IGCR1 enhancers upstream of Cm mostly promote V(D) J recombination [3, 4], while IgH 3′ regulatory region (3′RR) enhancers (hs3a, hs1,2, hs3b and hs4) promote SHM [5] and CSR [6] but not V(D)J recombination [7]. The 3′RR stimulates IgH transcription at the mature B cell stage [6]. The 3′RR is, thus, considered as a potential lymphoma oncogene deregulator. Strengthening this issue, transgenic mice models demonstrate the 3′RR implication in the development of several B cell lymphomas such as Burkitt lymphomas, mantle cell lymphomas and anaplastic B cell lymphomas [8-15] leading to the conclusion that the 3′RR might be a potential target for anti-lymphoma pharmacological therapy. Such approach would be promising if targeting the 3′RR does not induce adverse effects such as altered normal immune and inflammatory responses. Until now, the role of the 3′RR in B cell-mediated inflammatory responses is unknown. To test this issue we investigated the impact of the total 3′RR deletion on the well-known in *vivo* pristane-induced inflammatory response. The pristane is a powerful and very long acting immunological adjuvant in BALB/c mice. The *i.p.* pristane injection induces the development of a chronic inflammatory response with an influx of T cells, B cells and granulocytic cells into the peritoneal cavity as well as the development of ascites; functional B cells being critical for all these events [16, 17].

## RESULTS

### Generation of 3′RR-deficient BALB/c mice

**Figure 1:**
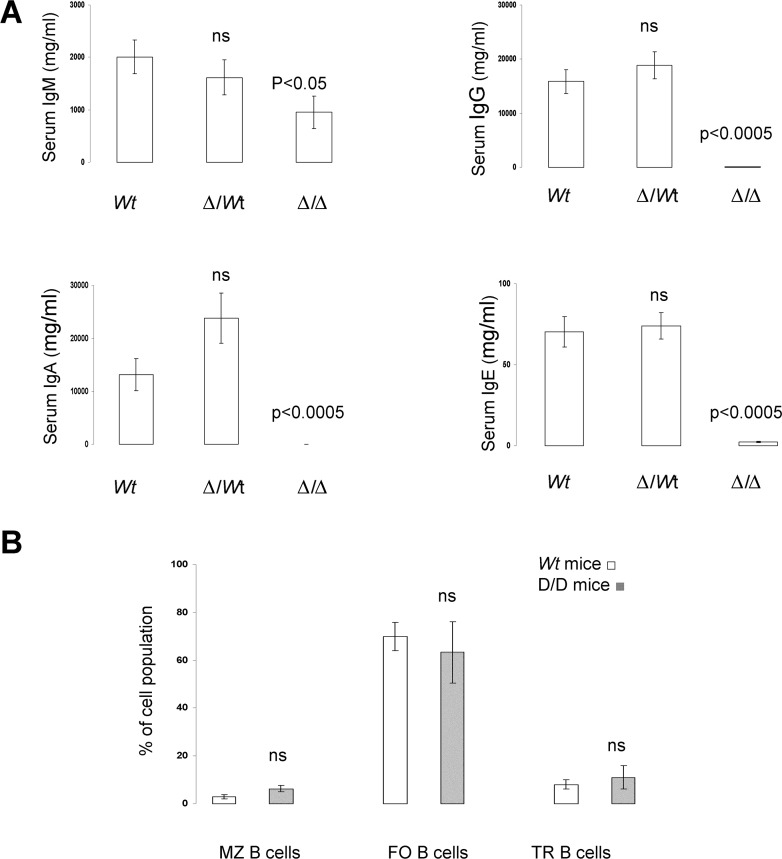
Serum Ig levels and B splenocyte phenotypes in 3′RR-deficient BALB/c mice A: Serum IgM, IgG, IgE and IgA were investigated in six 3′RR-deficient BALB/c mice (Δ/Δ), eighteen heterozygous Δ3′RR/*wt* BALB/c mice (Δ/*wt*), and sixteen *wt* BALB/c mice (*wt*). Significances (as compared with *wt* mice) were investigated by Mann-Whitney *U*-test. ns: not significant. All samples were appropriately diluted before each assay and data were corrected by the dilution factor. B: Flow cytometry analysis of the percentages of marginal zone (MZ, B220^+^CD21^+^CD23^−^) B cells, follicular (FO, B220^+^CD21^−^CD23^+^) B cells and transitional (TR, B220^+^CD21^−^CD23^−^) B cells in 3′RR-deficient BALB/c mice and *wt* BALB/c mice (3 months old). Mean ± SEM of 3 experiments. ns: not significant as compared with *wt* mice.

Although some phenotypes due to specific mutation are independent of the genetic background, phenotypic variability often becomes apparent only when a given mutation is studied on various genetic backgrounds [18- 21]. The 3′RR deletion, done in a 129 ES cell line and developed in a C57BL/6 background, was thus established in a BALB/c background. Similarly to that found in 3′RR-deficient C57BL/6 mice [6], 3′RR-deficient BALB/c mice had similar numbers of bone marrow, spleen and circulating B cells than *wt* BALB/c mice (data not shown). As previously reported in 3′RR-deficient C57BL/6 mice [6], 3′RR-deficient BALB/c mice showed a dramatic reduction (p<0.0005, Mann-Whitney *U*-test) for their serum IgG (84% of decrease), IgA (95% of decrease) and IgE (97% of decrease) levels (Figure 1A). Their serum IgM levels were reduced (p<0.05, Mann-Whitney *U*-test) to a lower extend (52% of decrease). Similarly to 3′RRdeficient C57BL/6 mice, their B splenocytes had a lower (p=0.008, Mann-Whitney *U*-test) capacity to secrete IgM (71.4 ± 11.2 ng/ml, mean ± SEM of 4 mice) after 3-days LPS stimulation *in vitro* than *wt* BALB/c mice (415.2 ± 140.4 ng/ml, mean ± SEM of 4 mice). Heterozygous Δ3′RR/*wt* BALB/c mice had similar serum levels of IgM, IgG, IgA and IgE than *wt* BALB/c mice (Figure 1A) confirming results with 3′RR-deficient C57BL/6 mice. Deletion of the 3′RR had no impact on the percentage of splenic marginal zone (MZ), follicular (FO) and transitional (TR) B cells (Figure 1B). Taken together these results indicate that the 3′RR deletion induces globally the same phenotypic pattern in C57BL/6 and BALB/c backgrounds.

### Generation of peritoneal ascites in 3′RR-deficient BALB/c mice

**Figure 2:**
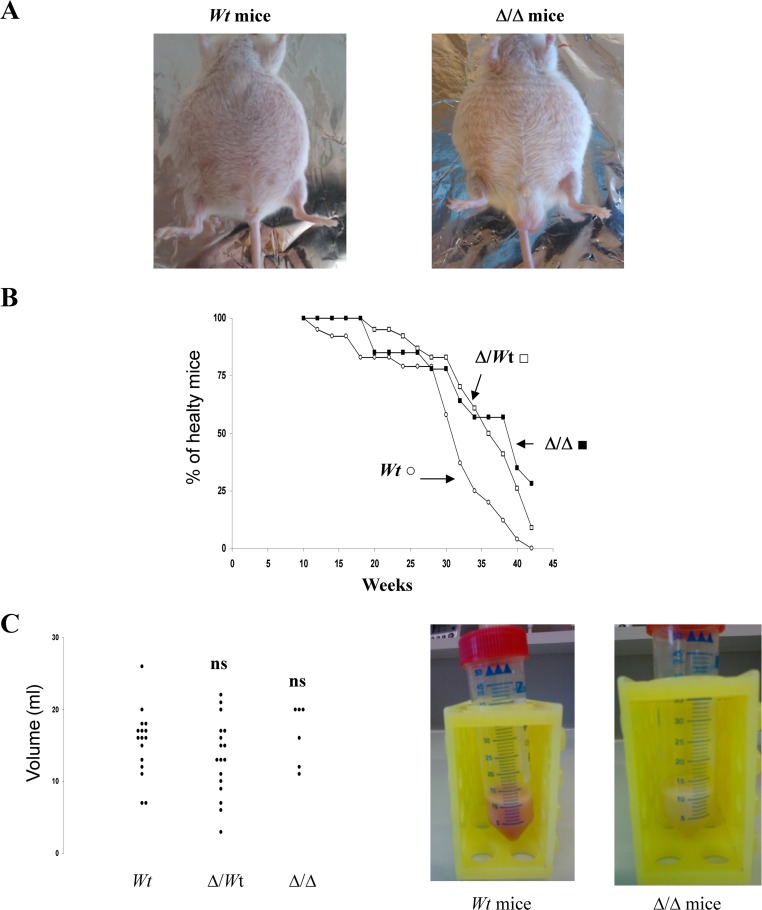
Ascite formation in 3′RR-deficient BALB/c mice A: Several months after pristane injection the development of a peritoneal ascite can be observed in *wt* BALB/c mice and 3′RR-deficient BALB/c mice (Δ/Δ). B: Fourteen 3′RR-deficient BALB/c mice (Δ/Δ), twenty one heterozygous Δ3′RR/*wt* BALB/c mice (Δ/*wt*) and twenty three *wt* BALB/c mice (*wt*) were treated with pristane and followed over a period of 10 months for peritoneal ascite development. C: Ascite volumes (in ml) obtained in Δ/Δ, Δ/*wt* and *wt* mice. ns: not significant as compared with *wt* mice (Mann-Whitney *U*-test). Pictures from peritoneal ascite samples from a *wt* mouse and a Δ/Δ mouse are presented.

About several months after pristane injection, development of ascites can be observed (Figure 2A). *Wt* BALB/c mice, Δ3′RR/*wt* BALB/c mice and homozygous 3′RR-deficient BALB/c mice were investigated. As shown in Fig. 2B, deletion of the 3′RR had no dramatic effect on the time course of ascite formation. If a full penetrance (100% ascite incidence) was fond in *wt* mice (23/23), theses values were of 90% (19/21) and 71% (10/14) for heterozygous and homozygous 3′RR-deficient BALB/c mice, respectively. The volumes of peritoneal exudates varied between mice (Figure 2C) but additional symptoms were always present such as lethargy, ruffled coats, enlarged abdomen and restricted gait [22]. Peritoneal exudate volumes were no significantly different (p>0.05, Mann-Whitney *U*-test) in *Wt* BALB/c mice (13.5 ± 1.6 ml, mean ± SEM of 16 mice), Δ3′RR/*wt* BALB/c mice (10.4 ± 1.6 ml, 17 mice) and homozygous 3′RR-deficient BALB/c mice (14.8 ± 2.4 ml, 6 mice) (Figure 2C). Mice with no ascite had normal viscera with clear peritonea and mesenteric tissue (data not shown).

### Leukocyte infiltrate in the exudates of pristaneinjected mice

**Figure 3:**
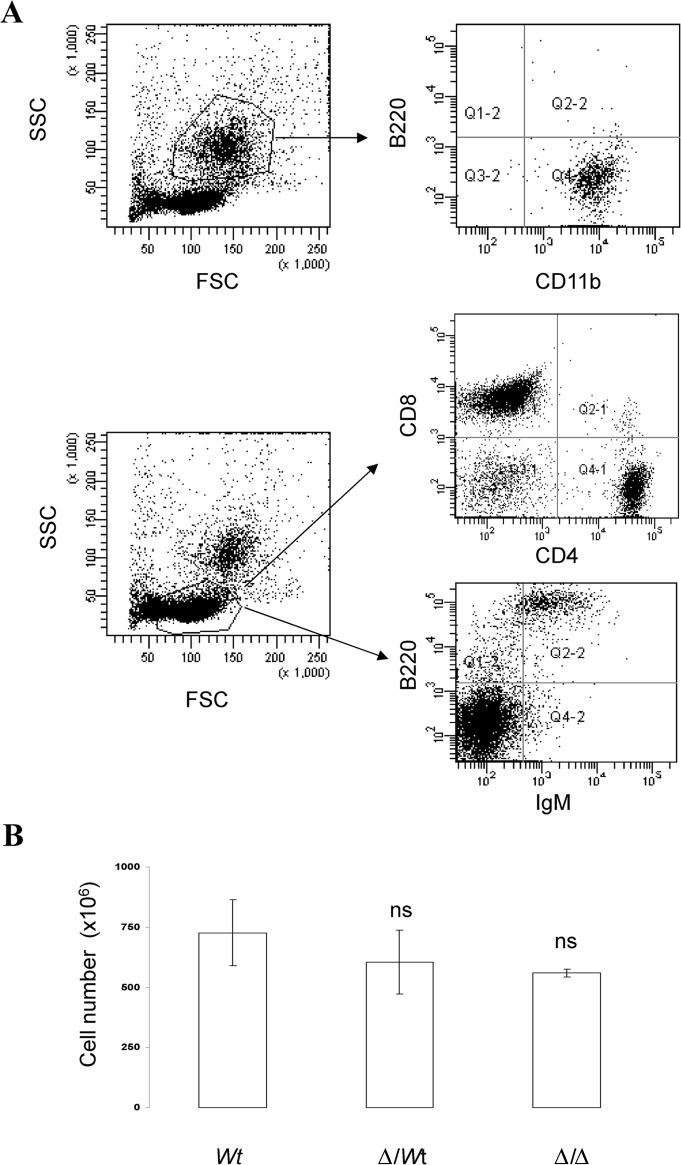
Leukocyte infiltrate in the exudates of pristane-injected mice A: Flow cytometry analysis indicated that granulocytic cells (CD11b^+^ cells), T lymphocytes (CD4^+^ and CD8^+^ cells) and B lymphocytes (B220^+^ cells) were the major cell species found in the peritoneal exudates of pristane-injected mice. A representative experiment is shown. B: Total cell number in the peritoneal exudates of pristane-injected mice. Mean ± SEM of three 3′RR-deficient BALB/c mice, twelve heterozygous Δ3′RR/*wt* BALB/c mice, and five *wt* BALB/c mice. ns: not significant (Mann-Whitney *U*-test) as compared with *wt* mice.

Flow cytometry analysis indicated that granulocytic cells (CD11b^+^ cells), T lymphocytes (CD4^+^ and CD8^+^ cells) and B lymphocytes (B220^+^ cells) were the predominant cell types in the exudates of pristaneinjected mice (Figure 3A) with no differences between *Wt* BALB/c mice, heterozygous Δ3′RR/*wt* BALB/c mice and homozygous 3′RR-deficient BALB/c mice. For T cells, no differences were documented for percentages of CD4^+^ and CD8^+^ cells. For B cells, no differences were documented for percentages of IgM^+^, IgD^+^, CD5^+^, CD43^+^ and CD138^+^ cells (Table 1). Finally, the total infiltrated cell numbers were not significantly different (p>0.05, Mann-Whitney *U*-test) for the three mouse categories (Figure 3B).

**Table 1:** Cellular composition in the peritoneal exudates of pristane-treated mice

	*Wt* mice (n=19-23)	Heterozygous Δ3′RR/*wt* mice (n=19-20)	3′RR-deficient mice (n=9-10)
CD11b^+^ cells	39.88 ± 4.03%	43.97 ± 5.79%	37.87 ± 6.70%
Total T cells	50.03 ± 3.14%	47.87 ± 4.87%	47.20 ± 7.14%
CD4^+^ T cells	66.28 ± 1.93%	64.23 ± 3.35%	62.49 ± 2.69%
CD8^+^ T cells	33.72 ± 1.93%	35.24 ± 3.12%	37.52 ± 2.69%
Total B cells	9.25 ± 0.88%	8.24 ± 1.30%	11.19 ± 2.0%
B220^+^IgM^+^ cells among B220^+^ cells	59.50 ± 3.20%	61.34 ± 4.53%	69.61 ± 4.06%
B220^+^IgD^+^ cells among B220^+^ cells	67.12 ± 2.88%	67.25 ± 2.87%	68.85 ± 4.71%
B220^+^CD5^+^ cells among B220^+^ cells	30.57 ± 2.27%	33.14 ± 2.59%	30.92 ± 4.18%
B220^+^CD43^+^ cells among B220^+^ cells	50.63 ± 4.42%	55.81 ± 4.76%	65.08 ± 4.04%
B220^+^CD138^+^ cells among B220^+^ cells	17.98 ± 4.66%	9.03 ± 1.11%	10.02 ± 3.04%
Total CD138^+^ cells	2.28 ± 0.63%	1.49 ± 0.39%	1.69 ± 0.53%

Cells were recovered, labelled with specific antibodies and analysed by flow cytometry. Results are reported as the mean ± SEM of the indicated number (n) of experiments. No significant differences (p>0.05) were found (Mann- Whitney *U*-test) between groups.

### Pro- and anti-inflammatory cytokines in the exudates of pristane-injected mice

We next investigated if deletion of the 3′RR altered the cytokine network in the exudates of pristaneinjected mice. Six cytokines with potent pro- and antiinflammatory effect were investigated: IL-6 [23], IL-21 [24], IL-12/23 [25], IL-10 [26, 27], interferon-γ [27] and TNF-α [28]. As shown in Table 2, similar TNF-α, IL-6, IL-21 and IL-12/23 levels (considered as proinflammatory compounds) were found in the exudates of pristane-injected 3′RR-deficient BALB/c mice, heterozygous D 3′RR/*wt* BALB/c mice and *wt* BALB/c mice. In contrast, IL-10 and INF-γ levels (considered as anti-inflammatory compounds) were slightly higher (p<0.05, Mann-Whitney *U*-test) in pristane-injected 3′RR-deficient animals.

**Table 2:** Cytokine and Ig levels in the peritoneal exudates of pristane-treated mice

	*Wt* mice (n=16)	Heterozygous Δ3′RR/*wt* mice (n=17)	3‘RR-deficient mice (n=6)
IL-6 (ng/ml)	5.52 ± 1.02	6.18 ± 1.21	5.55 ± 0.83
IL-12/23 (ng/ml)	2.42 ± 0.60	2.44 ± 0.23	2.65 ± 0.33
IL-21 (pg/ml)	17.50 ± 5.10	29.16 ± 5.60	21.60 ± 9.50
TNF-α (pg/ml)	103.5 ± 11.17	113.01 ± 11.68	109.0 ± 40.30
IL-10 (ng/ml)	0.39 ± 0.15	1.02 ± 0.21 (p<0.05)	1.21 ± 0.05 (p<0.05)
INFγ (pg/ml)	63.00 ± 9.32	94.4 ± 11.98	569.1 ± 400.56 (p<0.05)
IgM (mg/ml)	0.60 ± 0.06	0.63 ± 0.11	0.41 ± 0.12
IgG (mg/ml)	28.00 ± 2.88	29.72 ± 3.12	2.11 ± 0.41 (p<0.0005)
IgE (μg/ml)	21.94 ± 1.93	22.84 ± 1.83	0.05 ± 0.01 (p<0.0005)
IgA (mg/ml)	19.12 ± 3.28	21.20 ± 4.07	0.65 ± 0.14 (p<0.0005)

Cytokine and Ig levels were investigated with specific ELISA. Results are reported as the mean ± SEM of the indicated number (n) of experiments. Statistical significance (as compared with *wt* mice) was made with the Mann-Whitney *U*-test. All samples were appropriately diluted before each assay and data were corrected by the dilution factor.

### Ig levels in the exudates of pristane-injected mice

As shown in Table 2, Ig levels were dramatically lowered (p<0.0005, Mann-Whitney *U*-test) in the exudates of pristane-injected 3′RR-deficient BALB/c mice as compared with *wt* BALB/c mice with a 92%, 99% and 96% of decrease for IgG, IgA and IgE, respectively. In contrast, Ig levels were similar (p>0.05) for heterozygous D3′RR/*wt* BALB/c mice and *wt* BALB/c mice (Table 2). These results demonstrated the reduced capability of 3′RR-deficient B cells to efficiently switch toward IgG, IgE and IgA *in vivo* and thus to secrete switched Ig. In contrast, and in agreement with the fact that 3′RR deficiency did not impede plasma cell differentiation [6], IgM levels were not affected in 3′RR-deficient BALB/c as compared with *wt* BALB/c.

## DISCUSSION

Although some phenotypes due to specific mutations are independent of the genetic background, phenotypic variability often becomes apparent only when a given mutation is studied on various genetic backgrounds [18-21]. Specifically for the 3′RR, c-*myc*-3′RR transgenic mice developed Burkitt lymphomas in a C57BL/6 background but not in a BALB/c background [21]. Results of the present study indicate that the 3′RR deficiency induces similar phenotypic effects in a C57BL/6 and a BALB/c background: no effect on immature B cell stages, no impact on mature spleen B cell number, no impact in TR, FO and MZ B cell number, no effect on plasma cell differentiation but a severe CSR defect toward all isotypes resulting in depressed secretion of all Ig including IgM. If data report differences on spleen versus peritoneal B cell responses [22], similar impact of the 3′RR deletion is found for their switching and Ig secreting ability. Whether the 3′RR is considered as a potential lymphoma oncogene deregulator [8-15], no studies have focussed on its role in inflammatory processes. Do 3′RR-deficient B cells capable of sustaining efficient inflammatory and immune responses? We have, thus, investigated the impact of the total 3′RR deletion on the well-known in *vivo* pristaneinduced inflammatory response. The lack of 3′RR only marginally impacted (lower incidence) the development of ascite formation. When ascites were present, their cellular compositions and their kinetics of formation were similar to that found in *wt* mice. Among the different tested cytokines, pro-inflammatory cytokine levels (IL-6, IL-21, IL-12/23, TNF-α) were unchanged. In contrast, anti-inflammatory cytokine levels (IL-10, interferon-γ) were slightly increased in ascites of 3′RR-deficient animals showing an elevated anti-inflammatory reaction in 3′RR-deficient mice that might explain the lessened ascite development incidence. Clearly if the conventional versus pathogen free microenvironment status of mice is of importance for development of ascite formation [17], it is not the case for enhancers of the 3′RR. Our results clearly indicate that the 3′RR is dispensable for the efficient recruitment of immune cells in pristane-induced inflammation and has minimal impact on inflammatory responses. Moreover, the ability of IgM^+^ B cell to switch toward γ, ε and α isotypes and, thus, to secrete IgG, IgE and IgA is also dispensable to the development of an efficient pristane-induced inflammation.

In conclusion, if the 3′RR is considered as a major lymphoma oncogene deregulator [8-15, 29], its deletion has no dramatic effect on immune and inflammatory responses in the pristane mouse model. It is, thus, tempting to speculate that the 3′RR might be considered as a potential suitable target for anti-lymphoma pharmacological therapy without significant impact on the normal immune and inflammatory networks. A limitation of the pristane mouse model is that inflammation is restricted to the peritoneal cavity. It is of evidence that other mouse models of inflammatory reactions must be tested before definitive validation of this hypothesis.

## MATERIALS AND METHODS

### Generation of transgenic mice

Our research has been approved by our local ethics committee review board (Comité Régional d’Ethique sur l’Expérimentation Animale du Limousin, Limoges, France) and carried according the European guidelines for animal experimentation. The 3′RR deletion has been done in a 129 ES cell line and developed in a C57BL/6 background [6]. 3′RR-deficient C57BL/6 male mice were thus crossed with female BALB/c mice. The resulting male progeny were backcrossed with female BALB/c for more than six generations. The presence of the 3′RRdeleted allele was verified by PCR. 3′RR-deficient BALB/c mice, heterozygous Δ3′RR/*wt* BALB/c mice and *wt* BALB/c mice were investigated.

### PCR

PCR experiments for detection of the *wt* 3′RR allele were carried out with specific forward 5′-CCAAAAATGGCCAGGCCTAGG-3′ and reverse 5′-GACCCTGTCCTATGGCT GAC-3′ primers. PCR experiments for detection of the deficient 3′RR allele were carried out with specific forward 5′-TCCCTGGACAATCTGCACAT-3′ and reverse 5′-GACCCTGTCCT ATGGCTGAC-3′ primers. DNA was denatured 180 sec at 95°C, and then submitted to 35 cycles consisting in 94°C/30 sec, 60°C/30 sec and 72°C/60 sec. Amplification products were analysed on a 1.2% agarose gel. Expected sizes of amplified products were 250 bp and 587 bp for mutated and *wt* alleles, respectively.

### Inflammatory reaction induced by pristine

8-weeks-old mice were treated with three 0.5 ml *i.p.* injections of pristane on day 0, 15 and 30. According to the French law for animal experimentations, mice were sacrificed after the first sign of illness for example enlarged abdomen as a characteristic of accumulation of peritoneal exudates. Mice were followed over a period of 8 months.

### Flow cytometry analysis

Single-cell suspensions from spleen, bone marrow and cells of the peritoneal exudates were labelled with various antibodies (Southern Biotechnologies) and analyzed on a Fortessa LSR2 (Beckman Coulter).

### Blood and peritoneal exudates sampling

Blood samples were recovered from mice at the day of sacrifice. Serum samples were recovered by centrifugation and stored at – 20°C until used. After sacrifice, the peritoneal exudates were collected, centrifuged and stored at – 20°C until used.

### ELISA assays

Sera and peritoneal exudates were analysed for the presence of the various immunoglobulin (Ig) classes (IgM, IgG, IgE and IgA) by ELISA as previously described [8, 18]. IL-6, IL-10, IL-12/23, IL-21, TNF-α and INF-γ levels were determined in peritoneal exudates using specific ELISA (e-Bioscience). All samples were appropriately diluted before each assay and data were corrected by the dilution factor. Repeated ELISA determinations were submitted to a statistical analysis by using the Mann- Whitney *U*-test.

### Spleen cell cultures for IgM production

Single-cell suspensions of spleen cells were cultured 3 days at 1×10^6^ cells/ml in RPMI 1640 with 10% fetal calf serum and 20 mg/ml LPS. Supernatants were then harvested and stored at – 20°C until IgM evaluations as reported above [8, 30].
